# Improved Li–S Battery Performance with Dispersant/Plasticizer Co-Assisted Modification of a Poly(ethylene Oxide)/Li_6.4_La_3_Zr_1.4_Ta_0.6_O_12_ Solid Electrolyte

**DOI:** 10.1021/acsami.5c00987

**Published:** 2025-05-19

**Authors:** Ai-Yin Wang, Chun-Han Kuo, Yi-Chen Weng, Hao-Yu Liu, Chien-Hao Yeh, Yen-Lin Chen, Shu-Yu Chen, Hui-Ching Chien, Han-Yi Chen

**Affiliations:** 1 Department of Material Science and Engineering, 34881 National Tsing Hua University, 101, Sec. 2, Kuang-Fu Road, Hsinchu 300044, Taiwan; 2 56089 National Chung-Shan Institute of Science and Technology, Taoyuan 32546, Taiwan

**Keywords:** lithium−sulfur batteries, composite electrolytes, succinonitrile, poly(ethylene oxide)s, Li6.4La3Zr1.4Ta0.6O12

## Abstract

Lithium–sulfur batteries (LSBs) have garnered considerable attention over the past decade due to their high specific capacity and energy density. However, the poor safety and polysulfide shuttle phenomenon associated with liquid LSBs have been widely criticized. Solid-state electrolytes have the potential to overcome these issues, but their lower ionic conductivity and nonideal electrode/electrolyte interface contact as compared with liquid electrolytes remain a challenge in all-solid-state LSBs (ASSLSBs). This study applies the untested method of introducing a combination of dispersant and plasticizer as a “co-assisted” additive. We develop a polymer/ceramic composite electrolyte by combining poly­(ethylene oxide)­s, Li_6.4_La_3_Zr_1.4_Ta_0.6_O_12_ ceramic powder, the dispersant pluronic (C_3_H_6_O·C_2_H_4_O)_*x*_ (F127), and the plasticizer succinonitrile (C_2_H_4_(CN)_2_) (SN). The dispersant F127 effectively prevents the aggregation of ceramic powders, whereas the plasticizer SN reduces the crystallinity of the composite polymer electrolytes and decreases the interface impedance, thereby enhancing the overall ion conductivity. The resulting composite electrolyte exhibits an ionic conductivity of 1.24 × 10^–4^ S cm^–1^ at room temperature, and when coupled with a commercial sulfur electrode, a high capacity of 1085 mA h g^–1^ is achieved. In addition, the batteries demonstrate a high capacity retention of 71% after 100 cycles at a current density of 0.2 C at room temperature, demonstrating considerable promise for ASSLSB applications.

## Graphical abstract

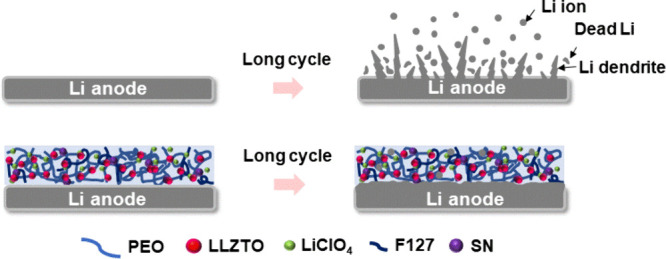

## 1 Introduction

With the rapid development of science and technology, electronic devices have become indispensable in our daily lives. However, the extensive use of cell phones and electric vehicles has led to high power consumption. Thus, the use of green energy is considered a good means of avoiding the worsening of global warming and protecting the environment. Recently, batteries with high energy and power densities have attracted increasing attention. Lithium-ion batteries (LIBs) are regarded as the most promising energy storage systems because of their high energy density, high output power, excellent charge–discharge cycle life, low self-discharge, and no memory effect.^1^ Among the different types of LIBs, those that employ sulfur or sulfur compounds as cathode materials and lithium metal as an anode material have attracted considerable attention. Lithium–sulfur batteries (LSBs) have extremely high theoretical capacity (1675 mA h g^–1^) and energy density (2800 W h L^–1^),^2^ which is 3–10 times higher than those of traditional LIBs.^3^ Sulfur materials are abundant on Earth and are inexpensive, nontoxic, and environmentally friendly. Therefore, LSBs are considered to be the most promising alternatives to traditional organic LIBs.

The cathode of LSBs is a composite material with sulfur or sulfide, and the anode is a lithium metal. The sulfur in the sulfur-composite material of the cathode produces several types of intermediates during the reaction. These intermediates are generally referred to as S_*n*_^2–^, such as S_8_^2–^, S_6_^2–^, S_4_^2–^, S_3_^2–^, and S_2_^2–^, and polysulfides such as S_8_^2–^, S_6_^2–^, and S_4_^2–^, which are easily soluble in the liquid electrolyte, causing a shuttle effect and reducing the discharge efficiency of LSBs.^4,5^

Despite their high theoretical capacity (1675 mA h g^–1^) and energy density (2800 W h L^–1^),^2^ LSBs still face several problems, such as poor electronic conductivity of sulfur (5 × 10^–30^ S cm^–1^)^6^ and large volume expansion of sulfur (80%) during lithiation, which leads to rapid decay in electrochemical performance. In addition, the polysulfide shuttle effect leads to continuous loss of active materials as well as a rapid decrease in capacity,^7^ and lithium metal dendrites cause internal short circuits and safety hazards.^8^ Among these problems, the polysulfide shuttle effect is the most severe as it directly affects the capacities and lifetimes of LSBs. Solid-state and quasi-solid-state electrolytes are considered feasible solutions to address these issues.^9,10^

Compared with volatile and flammable liquid organic electrolytes,^11–14^ solid electrolytes offer significantly improved safety.^15^ With no leakage risk, the manufacturing requirements for battery packaging made of solid electrolytes are lower than those for battery packaging made of traditional liquid electrolytes, resulting in lower costs. In addition, solid electrolytes effectively prevent the shuttle effect, loss of active materials, and a rapid decrease in electric capacity associated with sulfide dissolution into the electrolyte during charging and discharging. At room temperature, lithium ions have a high-performance migration number (*t*_Li+_ ≈ 1), allowing for uniform lithium deposition and inhibiting the formation of lithium dendrites, which can cause internal short circuits. Although solid electrolytes can effectively enhance battery safety and mitigate the polysulfide shuttle effect, a problem must still be overcome due to the generally poor compatibility between the solid electrolyte and electrode.^16^

To develop high-performance solid-state LSBs, the ideal solid-state electrolyte should possess the following characteristics: (1) high lithium-ion conductivity and migration number; (2) low electronic conductivity; (3) good mechanical strength; (4) larger potential range relative to electrode materials; (5) good chemical compatibility with cathodes and anodes; (6) excellent thermal stability; (7) low interfacial resistance at the electrode/solid electrolyte interface; and (8) low cost, safety, and environmental friendliness during production.^17^

Solid polymer electrolytes for LSBs are commonly prepared from poly­(ethylene oxide) (PEO) polymers. However, the ionic conductivity of solid electrolytes made from PEO and lithium salts is only 10^–7^–10^–6^ S cm^–1^ at room temperature. Previous studies have improved ionic conductivity by adding inorganic ceramic powders such as alumina and zirconium dioxide.^18^ In particular, the use of garnet-type ceramics such as Li_6.4_La_3_Zr_1.4_Ta_0.6_O_12_ (LLZTO) in composite electrolytes has shown promising advantages, including high lithium-ion conductivity and excellent electrochemical stability. Moreover, LLZTO can effectively suppress lithium dendrite growth and enhance the mechanical strength of the electrolyte.^19–21^ However, ceramic powder has a high surface energy, which makes it prone to particle agglomeration when mixed with a slurry. Adding a dispersant such as Pluronic (C_3_H_6_O·C_2_H_4_O)_*x*_ (F127) can result in more uniform dispersion of ceramic powder in solid ceramic electrolytes. F127 is a common dispersant formed by PEO and poly­(propylene oxide) (PPO) monomers, where its continuous carbon chain also transfers lithium ions, resulting in an ionic conductivity of 2.4 × 10^–3^ S cm^–1^.^22^ Succinonitrile (C_2_H_4_(CN)_2_) (SN) is a high-conductivity polymer electrolyte that is also used in artificial interfaces^23,24^ due to the high polarity of the carbon–hydrogen bonds that facilitate the dissociation of lithium salts. The chain orientational disorder and long-range translational order of SN contribute to excellent ionic conductivity (10^–3^ S cm^–1^) and a low melting point (57 °C).^25–28^

Numerous studies have explored the effects of dispersants on ceramic powder homogeneity in polymers and the role of plasticizers in reducing the overall crystallinity to enhance the ionic conductivity. However, the synergistic effects of these additives on solid electrolytes remain unknown. Herein, we propose a novel method that introduces dispersants and plasticizers, termed “co-assisted,” for fabricating a polymer/ceramic composite electrolyte using a tape-casting technique. Initially, the composite electrolyte was prepared by blending PEO, ceramic powder LLZTO, dispersant additive F127, and plasticizer SN. A doctor blade machine was used to obtain a thin and tightly bonded composite electrolyte for the cathode. As Figure 1 shows, the blue background represents the polymer electrolyte, and the light- and dark-blue lines correspond to PEO and F127, respectively. The black spheres represent the S@C composite materials, and the pink, green, and purple spheres represent LLZTO, LiClO_4_, and SN particles, respectively. By controlling the thickness of the coated electrolyte to 10 μm, we obtained a composite electrolyte that was thin and well bonded to the cathode. The resulting composite electrolyte exhibited an ionic conductivity of 1.24 × 10^–4^ S cm^–1^ at room temperature and, when coupled with a commercial sulfur electrode, a high capacity of 1085 mA h g^–1^ was achieved.

**Figure 1:**
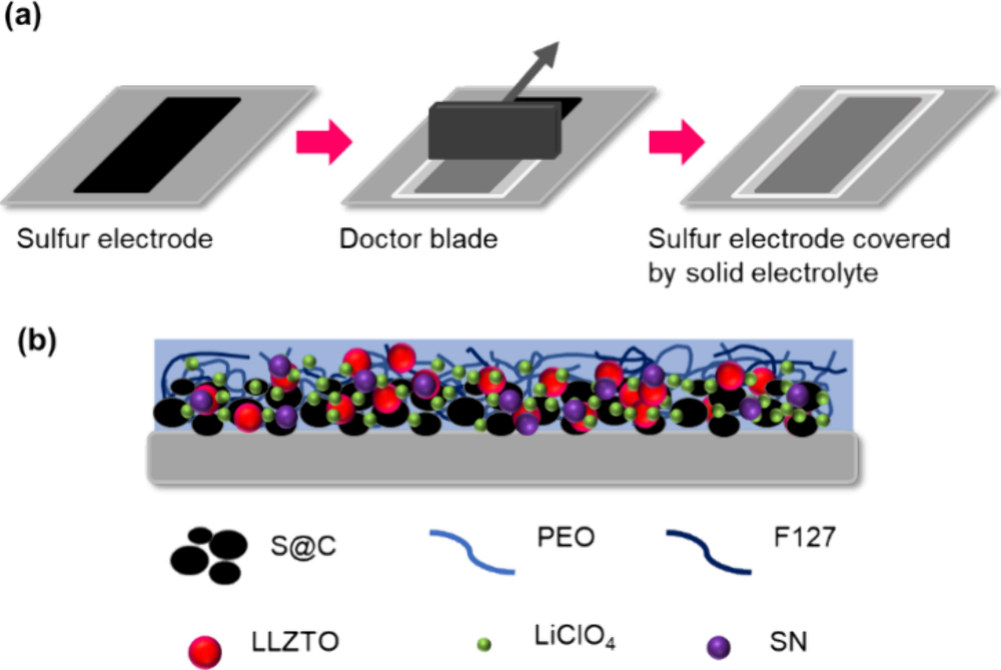
(a) Schematic of the composite electrolyte using the doctor blade method. (b) Cross-sectional view of the electrode structure.

## 2 Experimental Section

### 2.1 Preparation of a Composite Polymer Electrolyte

A precursor solution was prepared by mixing PEO (Sigma-Aldrich, Mw 600,000), dispersant pluronic (C_3_H_6_O·C_2_H_4_O)_*x*_ (F127) (Sigma-Aldrich), and solvent methanol under constant stirring at 60 °C. The lithium salt LiClO_4_(Acros Organics, 99+%), LLZTO powder (MTI CORP., >99.9%), and plasticizer succinonitrile (C_2_H_4_(CN)_2_) (SN) (Thermo Scientific, 99+%) were added to the solution and stirred for 30 min. The molar ratio of EO:Li was set to 16:1;^29,30^ prior to the main experimental procedure, the LLZTO content used in the investigation was tested by comparing the first discharge capacities of different LLZTO contents on PEO applied to the Li–S battery. As depicted in Figure S1, when the LLZTO content was 20 wt %, the Li–S battery demonstrated the best first discharge capacity and cycling stability. Accordingly, the LLZTO content was set to 20 wt %, the dispersant content to 10 wt % of LLZTO,^31–33^ and the plasticizer content to 33 wt % of the polymer and lithium salt.^34^ The obtained solution was sonicated for 1 h and subjected to 3D ball milling for 2 h. The solution was then sonicated for an additional 90 min to remove bubbles, and the resulting solution was used as the as-prepared composite polymer electrolyte (CPE) solution (denoted as CPE with F127/SN). The CPE without F127/SN was also prepared as a control, denoted as CPE w/o F127/SN. Optical images of the different solid electrolytes are presented in Figure S2.

### 2.2 Preparation of S@C Cathode and CPE-Based Quasi-Solid-State Li–S Batteries

S@C was prepared by using a hydrothermal method. Sulfur powder (Sigma-Aldrich, powder, ≥99.0%) (70 wt %) and Ketjen black (KB) (Ketjenblack EC-600JD) (30 wt %) were crushed and heated at 155 °C for 12 h to create the S@C composite. S@C material characterization is shown in Figure S3. Appropriate amounts of distilled water and ethanol were used to prepare a slurry containing 80 wt % S@C, 10 wt % carbon black, and 10 wt % acrylonitrile multicopolymer binder (LA133) (redox.me). The slurry was coated onto aluminum foil and dried in an oven for 24 h. The prepared CPE was coated evenly onto the S@C electrode using a 350 μm doctor blade and dried for 24 h to obtain the sample. The thickness of the electrolyte coating was approximately 10 μm.

Quasi-solid-state Li–S batteries were constructed using CR2032-type coin cells with electrodes having diameters of 14 mm (electrode area: 1.54 cm^2^). The cathode and anode of the coin cells were separated by using a CPE and polypropylene separator (Celgard 2500), with Li foil and S@C as the anode and cathode, respectively. A small amount (10 μL) of liquid electrolyte (0.5 M LiCF_3_SO_3_, 0.5 M LiNO_3_ in DME:DOL = 1:1 vol %) was used between the separator and the anode. Two spacers (1 mm each) were added to increase the pressure inside the battery to improve the performance. Coin cells were assembled in a glovebox filled with argon (H_2_O, O_2_ < 0.5 ppm).

### 2.3 Physicochemical Characterization and Electrochemical Performance of the CPE

The surface morphology of the CPE was analyzed by using field-emission scanning electron microscopy (8010 and JSM-IT800). Powder X-ray diffraction (XRD) analysis was conducted using a Bruker D2 Phaser instrument with Cu Kα radiation (wavelength λ = 1.5418 Å). Thermogravimetric analysis (TGA) was conducted employing a thermal analyzer (TG/DTA 6300, PerkinElmer) in an argon atmosphere at a flow rate of 60 mL min^–1^, spanning a temperature range of 25–800 °C at a heating rate of 5 °C min^–1^. A PerkinElmer Spectrum 2 Fourier-transform infrared spectrometer was used to obtain the FT-IR spectra. The ionic conductivities (S cm^–1^) of CPEs were determined using the following equation:^35^

$$\sigma =\frac{L}{RA}$$ 1

where L is the thickness of the CPE in micrometers (μm), A is the area of the electrode in square centimeters (cm^2^), and R is the resistance of the CPE in ohms (Ω). Electrochemical impedance spectroscopy (EIS) experiments were conducted using an electrochemical workstation (BioLogic SP300) with two stainless steel (SS) blocking electrodes sandwiched between the CPE in a symmetrical SS|CPEs|SS cell. The frequency range was 10.0 MHz to 10 mHz with an AC amplitude of 100 mV at room temperature (24 °C) to 100 °C. The Li^+^ transference numbers (*t*_Li+_) of the CPEs were determined using the Bruce–Vincent–Evans equation:^36^

$$t_{{\mathrm{Li}}^{+}}=\frac{I_{s}(\Delta V-I_{0}R_{0})}{I_{0}(\Delta V-I_{s}R_{s})}$$ 2

where Δ*V* is the polarization voltage (10 mV), *I*_0_ and *I_s_* are the initial (μA) and steady-state (μA) currents, and *R*_0_ and *R*_*s*_ are the initial (Ω) and steady-state (Ω) resistances, respectively. Galvanostatic cycling and linear sweep voltammetry (LSV) were performed by using an electrochemical potentiostat (BioLogic Potentiostat, VSP). To verify the electrochemical stability, LSV measurements were conducted on an asymmetric Li|CPEs|SS cell in the potential range between open-circuit voltage (with an on-chip variation of 2.1 V) and 5.5 V at a scanning rate of 1 mV s^–1^. For the galvanostatic cycling test, a symmetric Li|CPEs|Li cell was used and operated for over 350 h at a current density of 0.1 mA cm^–2^ and the surface capacity was 0.1 mA h cm^–2^. The cycle life of the quasi-solid-state Li–S batteries was evaluated by charging and discharging the cell at various currents between 1.7 and 2.8 V using a LANHE battery test system (CT2001A).

## 3 Results and Discussion

To investigate the effects of F127 as a dispersant and SN as a plasticizer on PEO, the crystallinity of the CPE was characterized by XRD. Figure 2a shows the XRD patterns of the different composite solid electrolytes, which were used to investigate the crystallinity of the CPEs before and after combining with F127 and SN. As Figure 2a shows, the PEO matrix has characteristic diffraction peaks at 2θ = 18.9° and 23.1°. After the formation of the CPE, the coordination interactions between the ether O atoms of PEO and Li^+^ caused an intensity difference in these diffraction peaks.^37^ As SN was introduced into the CPE, the intensities of these peaks became weaker and broader, indicating a decrease in the crystallinity of the PEO. This demonstrated the significant effect of SN as a plasticizer. A similar effect occurred when F127 and SN were both added to CPE. Notably, a single addition of F127 did not affect the crystallinity of the PEO. In addition to being an excellent plasticizer, SN is also an excellent ionizer of Li salts, releasing Li ions from LiClO_4_ to produce a significant increase in the amorphous state of the PEO matrix.^38^ The CPE with an SN or F127/SN mixture as an additive showed a decrease in crystallinity, which considerably improved the segmental mobility of the polymer chains. However, the effect of F127 has yet to be thoroughly understood and is elucidated in the morphological analysis of the SEM images in the following paragraph. Figure 2b shows a TGA thermogram of the different composite solid electrolytes. The CPE w/o F127/SN was found to be stable up to a final one-step degradation of approximately 410 °C. This may be due to the fact that the internal components were pure polymers, lithium salts, and ceramics. Thermal degradation of the CPE with F127/SN involved two steps. The first degradation step was due to the degradation of the plasticizer SN and the dispersant pluronic F127, which occurred at 105–265 °C, and another step was possibly due to the degradation of the polymer, which occurred at approximately 410 °C. Finally, the pure polymer volatilized almost completely, whereas the complexes left residual salt and ceramics. The CPEs exhibited stable performance in the operating temperature range of batteries, maintaining their integrity without decomposition or failure.

**Figure 2:**
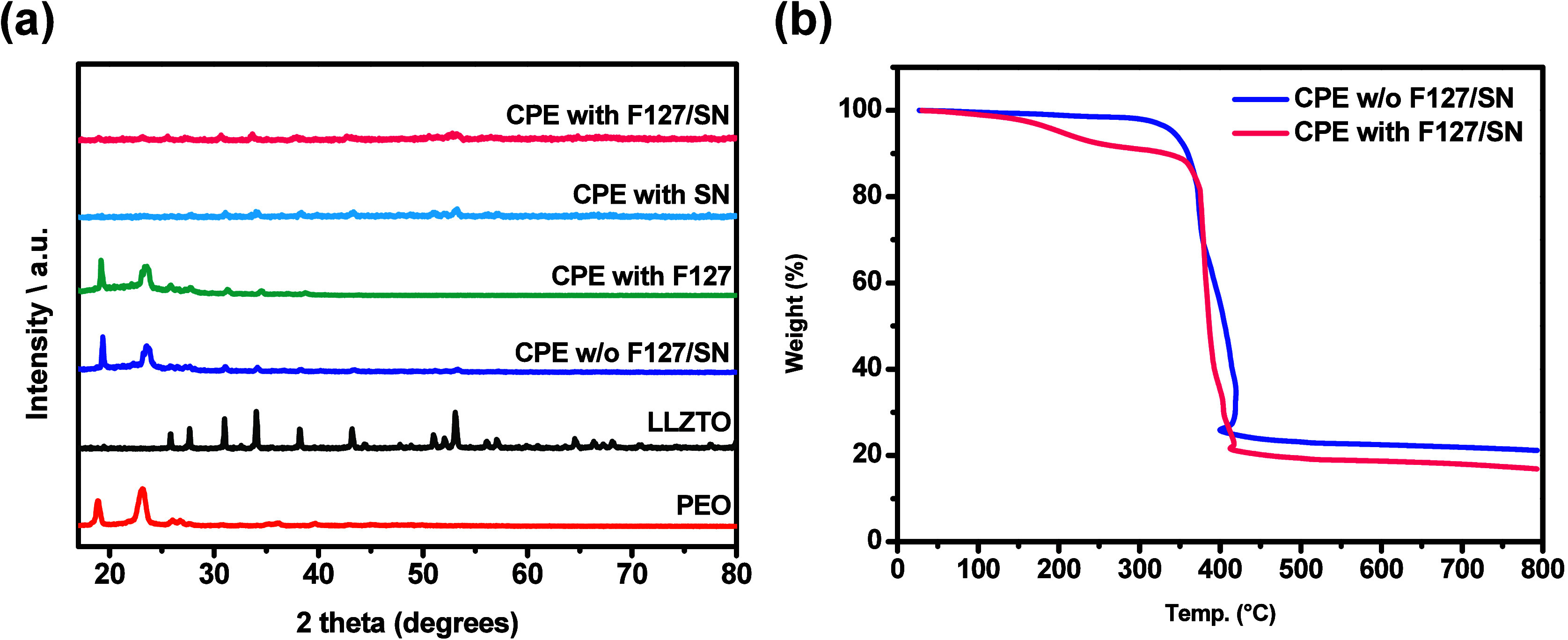
(a) XRD patterns of different composite solid electrolytes. (b) TGA thermograms of different composite solid electrolytes.

The Fourier-transform infrared spectroscopy (FT-IR) spectra of pure PEO, pure F127, Pure SN, and various CPEs are presented in Figure S4. For the PEO spectrum, the spectral bands observed at 2882 cm^–1^ correspond to C–H stretching vibrations, while those at 1467 and 1342 cm^–1^ are attributed to C–H bending vibrations. The absorption bands between 1280 and 1092 cm^–1^ arise from the stretching vibrations of the hydroxyl (O–H) group and the C–O–C ether linkage.^39^ F127 exhibits similar characteristic peaks. In the case of SN, a prominent absorption peak at 2254 cm^–1^ is observed, corresponding to the telescopic vibration of the cyano (−CN) group, confirming the presence of a strongly electron-withdrawing cyano group in the SN molecule.^38^ The vibrational frequencies of LiClO_4_, appearing around 625, 1090, and 1630 cm^–1^, are attributed to the internal vibrational modes of the ClO_4–_ anion.^40^ Changes in the spectral region between 1000–1140 cm^–1^ indicate interactions between the ether bonds and Li^+^ ions.^41,42^ The peak near 622 cm^–1^ observed in various CPEs can be assigned to free ClO_4–_ anions.^41^ Additionally, the intensity of the bands around 1468 cm^–1^ and 1360 cm^–1^, corresponding to the scissoring and wagging modes of CH_2_, respectively, decreases in the CPEs compared to pure PEO.^40^ When comparing the CPE with F127/SN to the CPE with F127/SN, the introduction of additives results in new absorption signals in the latter spectrum. These signals are absent in the individual spectra of F127 and SN, suggesting the formation of new binding types during composite formation. Notably, the spectral region between 1600–1800 cm^–1^ shows strong resemblance between the spectra of the CPE with F127/SN and the CPE with F127, while differing significantly from the CPE with SN. This indicates that F127 contributes to the modification of this region’s pattern. Since F127 and PEO contain the same monomer, their IR spectra are inherently similar. The observed changes in the 1600–1800 cm^–1^ region likely signify the formation of a complex yet reproducible cross-linkage structure between F127 and PEO, leading to a new bonding type absent in the original PEO or F127.

The SEM images and EDS mapping of the CPE with different additives covering the sulfur electrode are shown in Figure 3, where the EDS mappings were based on the region with higher magnification. A comparison of Figure 3b,a reveals that F127 demonstrated an influential dispersing phenomenon, and the morphology shows that the agglomeration phenomenon decreased significantly. A comparison of Figure 3c,a reveals that the morphology did not show a significant difference following the single addition of SN, which is reasonable because it does not possess any dispersant quality. A comparison of Figure 3d,c reveals the dispersion effect of F127, even though this effect was not as effective as that shown in Figure 3b. This was due to the partial effect of F127 (mixed with SN) as shown in Figure 3d, compared with the sole addition of F127 as shown in Figure 3b. Nevertheless, with the decreased crystallinity of the CPE with F127/SN and the dispersed particle morphology of the CPE w/o F127/SN, the improved electrochemical properties could be expected. The “co-assisted” effect on the dispersion of the particles or reduced crystallinity could be observed from both the XRD and SEM analysis.

**Figure 3:**
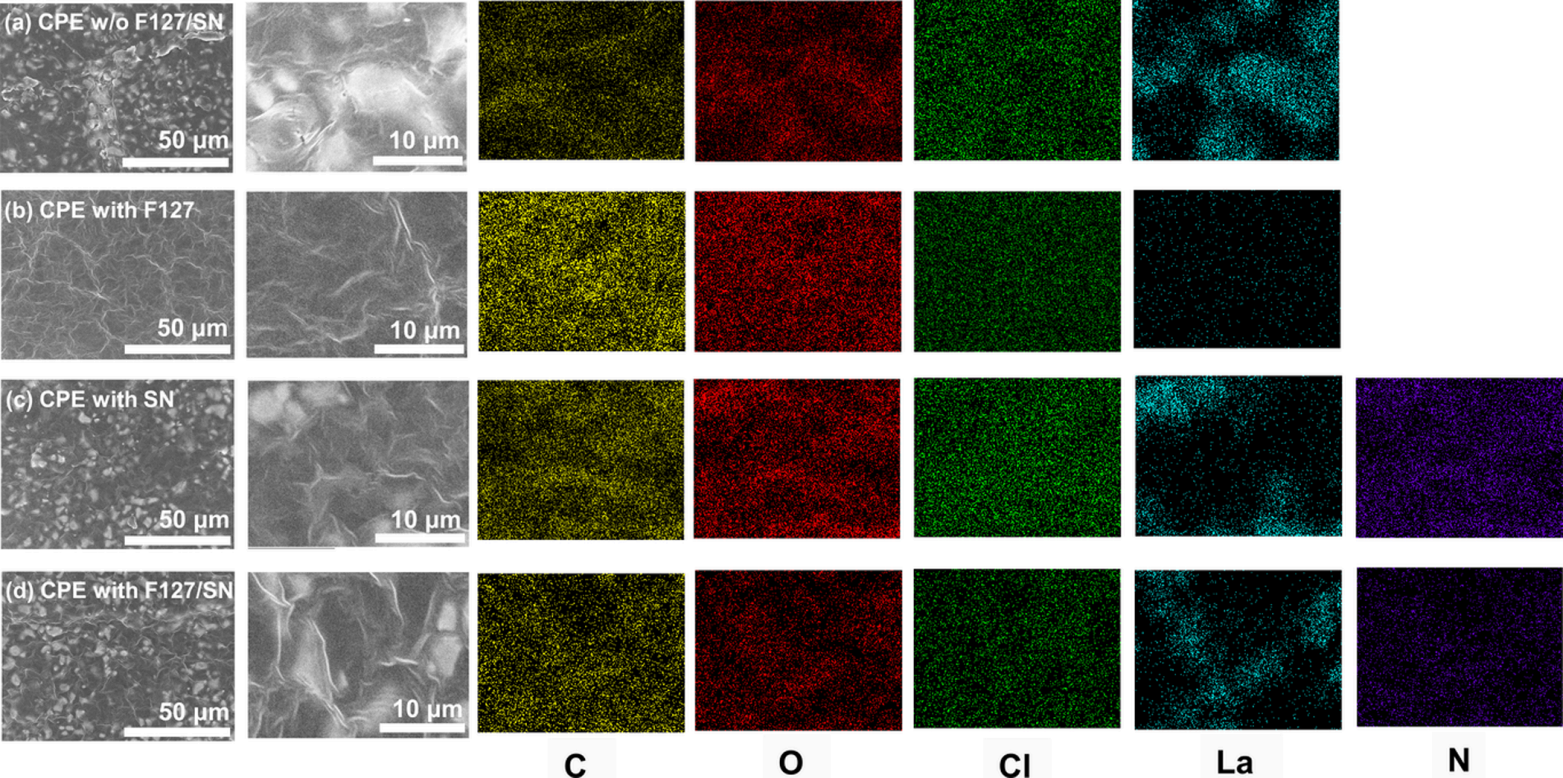
SEM images and EDS mapping of the different electrolytes: (a) CPE w/o F127/SN, (b) CPE with F127, (c) CPE with SN, and (d) CPE with F127/SN.

In addition to the dispersed morphology due to the introduction of F127, EDS mapping of the elements elucidated a similar phenomenon. The signals of carbon and oxygen originated mainly from the PEO, and the chlorine originated from LiClO_4_. These three elements showed good homogeneity in all treatments, as shown in Figure 3, indicating that LiClO_4_ has good dispersibility in the PEO. When we focus on the difference in the lanthanum mapping in each treatment, in which the signal originated from LLZTO, a comparison of Figure 3b,a or Figure 3d,c reveals the improved homogeneity of the lanthanum distribution, indicating the improved dispersion behavior of LLZTO when F127 was introduced. Due to the relatively weak signal in Figure 3b, additional elemental mappings obtained by EDS have been provided in Figure S5. A comparison of Figure 3d,c reveals the same scenario in terms of the distribution of nitrogen, which originated from the SN.

After confirming the effects of the additive on the crystallinity and morphology, we tested the CPE to verify that the transformation in the original contents was related to the kinetics and the electrochemical properties. Figure 4a displays the EIS curves of the CPE series. The equivalent circuit consisted of a resistor in series with an RC parallel combination, followed by another capacitor in series. R1 represents the contact resistance between stainless steel and the composite film. CPE1 represents the double-layer capacitance at the interface between stainless steel and the composite film. CPE2 and R2 represent the geometrical capacitance and bulk resistance of the composite polymer electrolyte, respectively. The ionic conductivity of the CPE w/o F127/SN, CPE with F127, CPE with SN, and CPE with F127/SN were 8.98 × 10^–6^, 1.52 × 10^–5^, 7.24 × 10^–5^, and 1.24 × 10^–4^ S cm^–1^, respectively. The EIS results revealed that introducing only F127 to reduce LLZTO agglomeration or only SN to reduce the crystallinity was both an effective method for increasing ionic conductivity, as this promoted faster lithium-ion transport. When the CEO was “co-assisted” by both F127 and SN, the combined effect of the dispersant and the plasticizer showed even better ionic conductivity than when either of these two was singly added to the CPE.^25^ Figure 4b illustrates how the conductivity of the composite electrolyte membranes based on PEO changed with temperature. With increasing temperature, a corresponding increase in conductivity was observed. This phenomenon was likely attributable to the increased structural relaxation observed at higher temperatures, which facilitated the release of more Li^+^ ions bound to the oxygen atoms of the PEO. In addition, increased temperatures tend to increase the mobility and frequency of charge carriers, thereby augmenting the overall ionic conductivity.^43^ The data points presented in Figure 4b are listed in Table 1.

**Figure 4:**
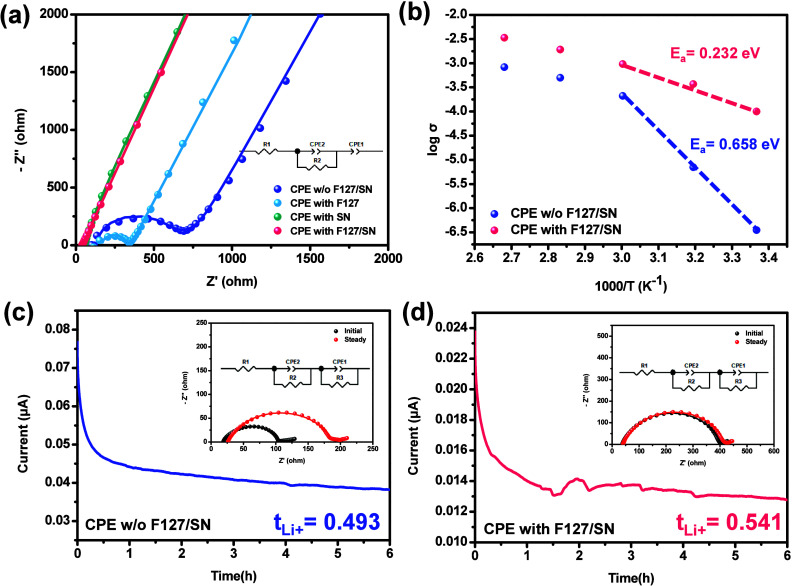
(a) EIS curves of different CPEs at room temperature in a symmetrical SS|CPEs|SS cell. (b) Arrhenius plots of the ionic conductivities of the CPEs. Chronoamperometry of Li|CPEs|Li cells at room temperature assembled with (c) CPE w/o F127/SN and (d) CPE with F127/SN. Inset: impedance spectra for the same cells before and after polarization.

**Table 1:** Ionic Conductivities of CPEs at Different Temperatures

sample	24 °C	40 °C	60 °C	80 °C	100 °C
CPE w/o F127/SN	3.50 × 10^–7^	7.00 × 10^–6^	2.10 × 10^–4^	5.00 × 10^–4^	8.30 × 10^–4^
CPE with F127/SN	1.00 × 10^–4^	3.70 × 10^–4^	9.60 × 10^–4^	1.92 × 10^–3^	3.36 × 10^–3^

Figure 4a,b shows that the introduction of both F127 and SN significantly affected the ionic conductivity of the CPE. The calorimetry findings revealed that the incorporation of SN significantly enhances the amorphous phase fraction, leading to a higher concentration of free charge carriers and resulting in increased conductivity.^25,44^ In addition, the graph presented in Figure 4b shows two distinct changes in the slope. Because the melting temperature of the crystalline PEO domain is approximately 60 °C,^45^ the segment below 60 °C was utilized to calculate its activation energy. The activation energy (Ea) for Li^+^ migration within these electrolyte membranes was determined using the Arrhenius equation $\sigma (T)=A\exp\;\frac{-Ea}{RT}$ .^46^ The CPE with F127/SN exhibited an Ea value of 0.232 eV, which was notably lower than that of the CPE without F127/SN (0.658 eV), indicating that ion transfer was facilitated in the CPE with the addition of F127/SN. Figure 4c,d shows the chronoamperometry profile, and the inset shows the EIS before and after the DC polarization test. R1 is the resistance of the film, R2 is the interfacial resistance, CPE2 is the contribution of the passivation layer (SEI) generated by the contact between the film and metal, R3 is the charge transfer resistance, and CPE1 is the resistance and capacitance effects associated with the movement of ions between the metal electrode and film.

Table 2 displays the lithium transference numbers, which were evaluated by using chronoamperometry and calculated for two CPEs. The *t*_Li+_ values were determined by eq 2, along with parameters from chronoamperometry and EIS tests for CPEs w/o F127/SN and with F127/SN; the values were 0.493 and 0.541, respectively, at room temperature. Adding F127/SN increased the number of migrating ions, which enhanced ionic conductivity. Increased cationic motion in lithium batteries can effectively mitigate polarization and hinder the formation of lithium dendrites.^47^

**Table 2:** Direct Currents (*I*_0_, *I*_*s*_), Charge Transfer Resistances (*R*_0_, *R*_*s*_) and Lithium Transference Numbers (*t*_Li+_) Estimated Using Equation 2 for Li|CPE|Li Cells

sample	*I*_0_ (μA)	*I*_s_ (μA)	*R*_0_ (Ω)	*R_s_* (Ω)	*t*_Li+_
CPE w/o F127/SN	0.077	0.038	84.64	160.57	0.493
CPE with F127/SN	0.024	0.013	397.07	416.62	0.541

LSV experiments were conducted to investigate the electrochemical window versus Li/Li^+^ for the CPEs, as shown in Figure 5a. The results demonstrated a wide electrochemical window of approximately 3.8 V versus Li/Li^+^, indicating its potential application in composite solid electrolytes for Li–S batteries. As Figure 5b shows, galvanostatic cycling tests were conducted on symmetric Li cells composed of CPE with F127/SN at room temperature. The detailed enlarged curve is presented in Figure S6. The overpotential of each cycle changed in the positive and negative halves, corresponding to Li dissolved from and deposited on the pellets, respectively.^48^ After the cell was cycled at a current density of 0.1 mA cm^–2^ for a few cycles, the CPE with F127/SN showed better stability against lithium metal than the CPE without F127/SN, indicating stable interfacial contact with the lithium metal. To further evaluate the stability of the symmetric cell, additional cycling tests were performed at varying current densities, as shown in Figure S7. The results align well with those obtained previously. These findings suggest that the CPE with F127/SN has promising potential for stable applications in Li–S batteries. To confirm this hypothesis, SEM was utilized to examine the surface structures of the CPEs after cycling. Figure 5c shows an SEM image captured prior to cycling of the lithium metal. Figure 5d,g shows the cycling profiles of the CPE with F127/SN and CPE without F127/SN, respectively. Figure 5i shows the lithium electrode of the symmetric cell of the CPE without F127/SN, showing conspicuous lithium dendrites and a fragmented surface. These characteristics may result in short circuits and heightened polarization. In contrast, the lithium electrode of the symmetric cell of CPE with F127/SN exhibited a uniform and dense surface devoid of lithium dendrites, as shown in Figure 5f. In addition, SEM images of the CPE surfaces after cycling were obtained. As shown in Figure 5e,h, noticeable changes were observed on the surface of the CPE without F127/SN. This is in contrast to the CPE with F127/SN, on the surface of which no significant changes were observed. This demonstrates that the film can maintain its structure after long-term cycling experiments, effectively resisting the generation of lithium dendrites and sustaining its overall functionality.

**Figure 5:**
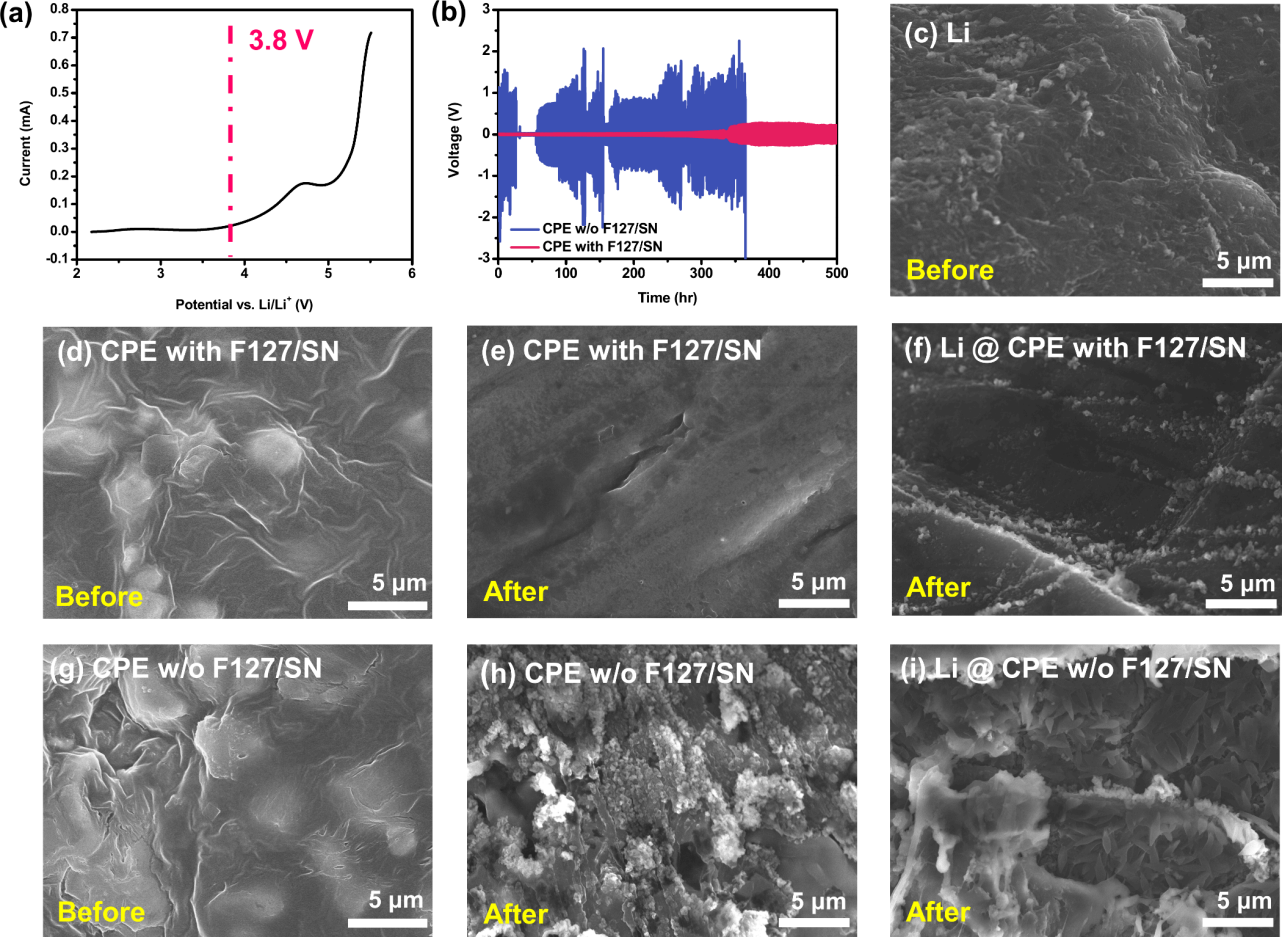
Stability tests of CPEs. (a) LSV of the CPE with F127/SN, and (b) galvanostatic cycling of the CPE with F127/SN and CPE w/o F127/SN. SEM images of (c) Li metal before cycling, (d) CPE with F127/SN before cycling, (e) CPE with F127/SN after cycling, (f) Li @ the CPE with F127/SN after cycling, (g) CPE w/o F127/SN before cycling, (h) CPE w/o F127/SN after cycling, and (i) Li @ CPE w/o F127/SN after cycling.

Figure 6 provides clearer explanations of the plasticizer and dispersant. After prolonged charging and discharging, conventional lithium batteries tend to develop lithium dendrites, leading to poor long-term cycling stability, as illustrated in Figure 6a. With the CPE w/o F127/SN, LLZTO aggregation enabled the formation of lithium dendrites, resulting in cycling stability below expectations, as shown in Figure 6b. By contrast, with the CPE with F127/SN, the dispersant F127 prevented LLZTO aggregation, ensuring uniform dispersion throughout the film’s corners and effectively inhibiting lithium dendrite growth, as depicted in Figure 6c. The SEM image of CPE with F127/SN after cycling is also shown in Figure S8. Moreover, the plasticizer SN promoted better adherence to the electrode surface,^49,50^ thus addressing the interface issues and thereby enhancing the overall cycling stability.

**Figure 6:**
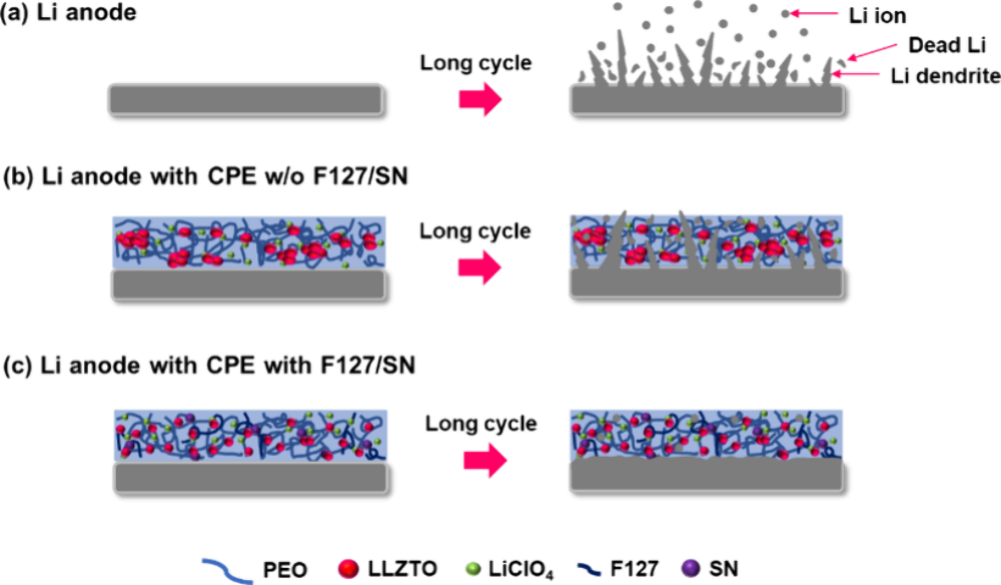
Lithium dendrite growth in (a) the Li anode, (b) the Li anode with CPE w/o F127/SN, and (c) the Li anode with CPE with F127/SN.

The electrochemical performances of the Li–S batteries with various CPEs are shown in Figure 7. Figure 7a shows the charge–discharge curve of a Li–S battery composed of different CPEs and S@C electrodes at a current density of 0.2 C at room temperature. In general, CPEs with three different compositions displayed similar charge–discharge behaviors. However, the discharge plateau of the battery with a liquid electrolyte was slightly higher than that with CPEs because of the more severe polarization in solid electrolytes.^51^ Figure 7b shows the charge–discharge curve of a Li–S battery composed of the CPE with F127/SN and S@C electrodes at room temperature. The initial activation discharge capacity was 2552 mA h g^–1^ at a current density of 0.05 C. However, it significantly dropped to 1085 mA h g^–1^ in the second cycle at a current density of 0.2 C. Similar phenomena could be observed in other research.^52–55^ The rapid fading of the irreversible capacity was due to the incomplete formation of the SEI layer in the initial cycle, and some sulfur particles were not fully activated, possibly due to sulfur spillover or agglomeration.^52,54,55^ However, after an activation cycle at a low current density, the charge–discharge behavior tended to be more stable.

**Figure 7:**
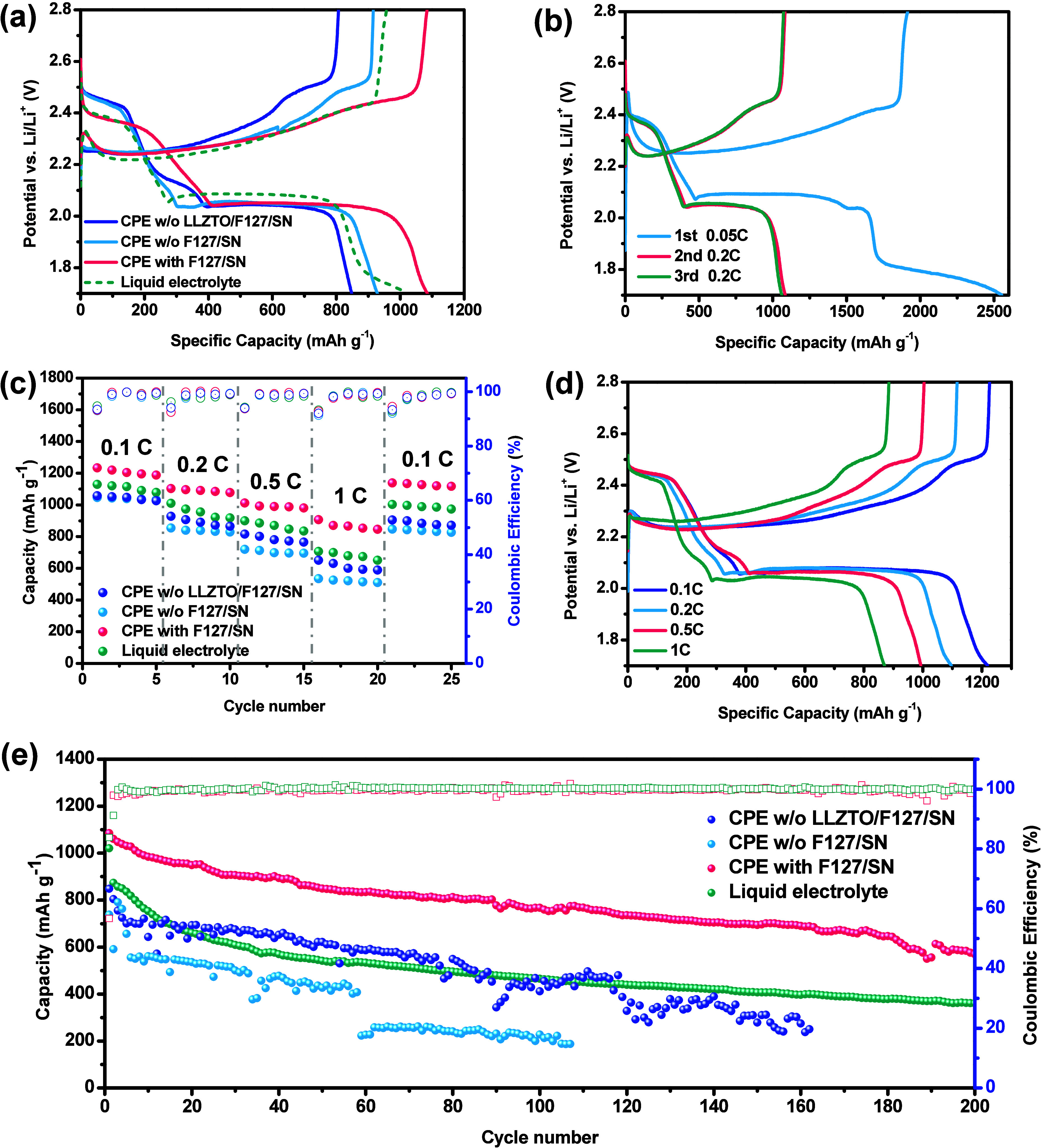
Electrochemical performances of Li–S batteries with different CPEs at room temperature. (a) Charge–discharge curves of Li–S batteries with different CPEs at a current density of 0.2 C. (b) Charge–discharge curves of the first to third cycles of a Li–S battery with CPE with F127/SN. (c) Rate performances of Li–S batteries with different CPEs. (d) Charge–discharge curves of Li–S batteries with CPE with F127/SN at different current densities. (e) Cycling stability and Coulombic efficiency of Li–S batteries with different CPEs at a current density of 0.2 C.

Figure 7c,d shows the rate performances and charge–discharge voltage curves of the lithium sulfur batteries prepared with different electrolytes. The rate performance was investigated at current densities of 0.1, 0.2, 0.5, and 1 C. Even at a high discharge rate, the battery built with the CPE with F127/SN showed a significantly larger capacity. The specific capacity reached 1218, 1096, 992, and 870 mA h g^–1^ under current densities of 0.1, 0.2, 0.5, and 1 C, respectively. With the dispersant of F127, the ceramic powder was well distributed in the polymer matrix. SN used as a plasticizer can reduce the crystallinity of the PEO. Thus, the battery had a better performance. In addition, the specific capacity remained stable during circulation at various ratios. To further investigate the kinetic performance of the different electrolytes during the charge–discharge process, GITT measurements at 0.1C were conducted, and the results are shown in Figure S9. It presents the galvanostatic intermittent titration technique (GITT) profiles of the liquid electrolyte, the CPE without F127/SN, and the CPE with F127/SN, all tested under comparable sulfur loadings, to evaluate the kinetic and thermodynamic benefits of incorporating F127/SN into the CPE. The GITT curves display the characteristic charge–discharge plateaus of Li–S batteries; notably, the CPE containing F127/SN exhibits more stable and higher plateau capacities with reduced polarization, indicating enhanced reaction kinetics.

Figure 7e shows the cycling stability and Coulombic efficiency of the Li–S battery composed of different CPEs and liquid electrolytes at a current density of 0.2 C at room temperature. The cell of CPE with F127/SN delivered an initial discharge capacity of 1085 mA h g^–1^ and, after 100 cycles, was reduced to 767 mA h g^–1^, corresponding to 71% capacity retention. After 200 cycles, it maintained a 53% capacity retention and its Coulombic efficiency was greater than 99.99%. Compared with other batteries composed of different CPEs and liquid electrolytes, they exhibited significant maintenance performance. This result demonstrated that the solid polymer electrolyte could prevent polysulfide from dissolving in the solution. For the CPE w/o F127/SN, the agglomeration of LLZTO particles hindered Li-ion transport, resulting in a worse performance. Compared with similar types of lithium–sulfur battery devices, as shown in Table 3, this study demonstrated competitive performance in terms of both energy capacity and long-term cycling stability. This represents a highly promising research endeavor.

**Table 3:** Composite Polymer Electrolytes for Li–S Batteries in Different Studies[Table-fn t3fn2]

electrolyte composition	additives	thickness (μm)	ionic conductivity (S cm^–1^)	cathode materials	capacity (mA h g^–1^)	cycling retention	ref.
**LLTO** with **PEO/LiTFSI**			1.5 × 10^–4^ @30 °C	S@C	1235 @60 °C @0.05C	73%_100th @60 °C @0.05C	^56^
**LLZTO** with **PEO/LiClO**_**4**_		20–75	9.5 × 10^–6^ @20 °C	S@LLZO@C	1050 @37 °C @0.05C	86%_200th @37 °C @0.05C	^57^
			1.1 × 10^–4^ @40 °C				
**LLZTO** with **PEGDA/LiTFSI**		200–400	1.3 × 10^–3^ @30 °C	S@CNT	1201 @25 °C @0.1C	70.2%_100th @25 °C @0.05C	^58^
			1.8 × 10^–3^ @70 °C				
**LLZTO** with **PVDF-HFP**	PAA/PSSLi/C2	100	3.7 × 10^–4^ @55 °C	S@C	935 @0.05C	64%_100th @0.05C	^32^
			5.6 × 10^–4^ @75 °C				
**LLZTO** with **PEO/LiClO**_**4**_	F127/SN	10–15	1.24 × 10^–4^@RT	S@C	1085 @RT @0.2C	71%_100th @RT @0.2C	this work

a LLZTO: Li_6.4_La_3_Zr_1.4_Ta_0.6_O_12_; SPE: solid polymer electrolyte; PEO: poly­(ethylene oxide); PEGDA: polyethylene glycol diacrylate; PVDF-HFP: poly­(vinylidene fluoride-*co*-hexafluoropropylene); PAA: poly­(acrylic acid); PSSLi: poly­(4-styrene sulfonic acid); C2: copolymer of succinic anhydride-modified epoxy-amine poly­[(propylene oxide)-*co*-(ethylene oxide)]; F127: pluronic (C_3_H_6_O·C_2_H_4_O)_*x*_; SN: succinonitrile (C_2_H_4_(CN)_2_).

## 4 Conclusions

We successfully synthesized a polymer/ceramic composite electrolyte by combining PEO, LLZTO, the dispersant additive F127, and the plasticizer SN by using a tape-casting process with a doctor blade. SEM images showed that the LLZTO particles were well distributed in the polymer matrix, and the addition of SN and F127 enhanced the Li-ion transport ability and reduced the interfacial resistance between the solid electrolyte and the electrode. The dispersant and plasticizer co-assisted composite electrolyte demonstrated high ionic conductivity of 1.24 × 10^–4^ S cm^–1^. In addition, we demonstrated that the composite electrolyte, when combined with commercial sulfur electrodes, produced a lithium–sulfur battery with a high capacity of 1085 mA h g^–1^ at a current density of 0.2 C at room temperature. After 100 cycles, the battery exhibited a high capacity retention of 71%. The results suggest that a CPE with F127/SN could be coated on a sulfur electrode by using a doctor blade to obtain a thin and high-performance lithium–sulfur battery. This battery has great potential for future applications in electronics.

## Supplementary Material



## Notes

Rationale for selecting 20% LLZTO, optical imaging of CPES, identification of cathode materials, FT-IR spectra, GITT analysis, and galvanostatic cycling results of the CPE, among other relevant data (PDF)
