# Supplementary material for: Improved Li–S Battery Performance with Dispersant/Plasticizer Co-Assisted Modification of a Poly(ethylene Oxide)/Li6.4La3Zr1.4Ta0.6O12 Solid Electrolyte
Source: ACS Appl Mater Interfaces. 2025 May 19;17(21):31334–44. doi: 10.1021/acsami.5c00987 (PMC12123572; doi:10.1021/acsami.5c00987)
Supplement: Supplementary file 1 [file am5c00987_si_001.pdf]

Supporting Information

Improved Li-S Battery Performance with Dispersant/Plasticizer Co-assisted Modification of a Polyethylene Oxide/$Li_{6.4}La_3Zr_{1.4}Ta_{0.6}O_{12}$ Solid Electrolyte

Ai-Yin Wang[1], Chun-Han Kuo[1], Yi-Chen Weng[1], Hao-Yu Liu[1], Chien-Hao Yeh[1], Yen-Lin Chen[1], Shu-Yu Chen[1], Hui-Ching Chien[2], and Han-Yi Chen[1,]*

1Department of Material Science and Engineering, National Tsing Hua University, 101, Sec. 2, Kuang-Fu Road, Hsinchu 300044, Taiwan

2National Chung-Shan Institute of Science and Technology, Taoyuan 32546, Taiwan

*Corresponding Author: hanyi.chen@mx.nthu.edu.tw (Prof. Han-Yi Chen)

Number of Pages: 5
Number of figures: 9
Number of tables: 0

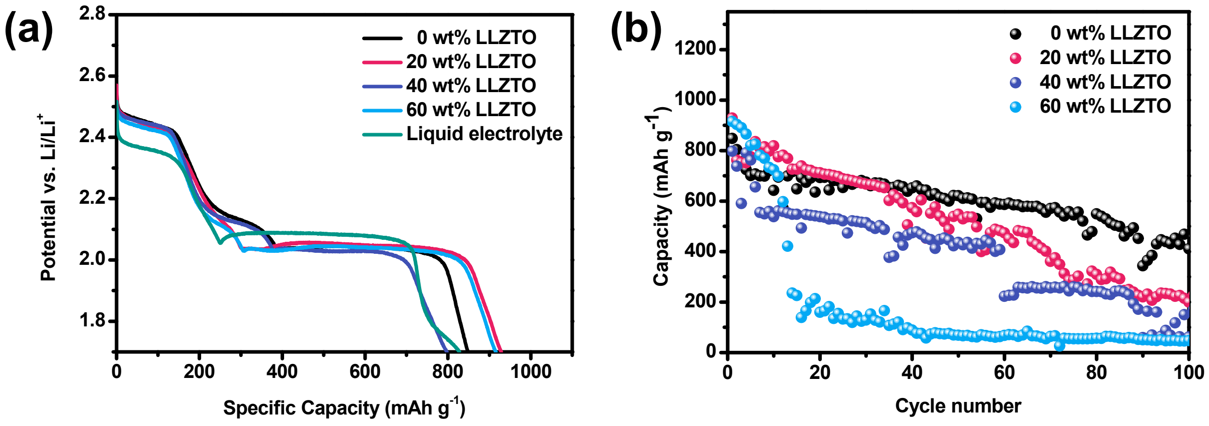

Figure S1 Li-S batteries with CPEs with different LLZTO ratio **(a)** Discharge curves (b) Cycling stability at a current density of 0.2 C

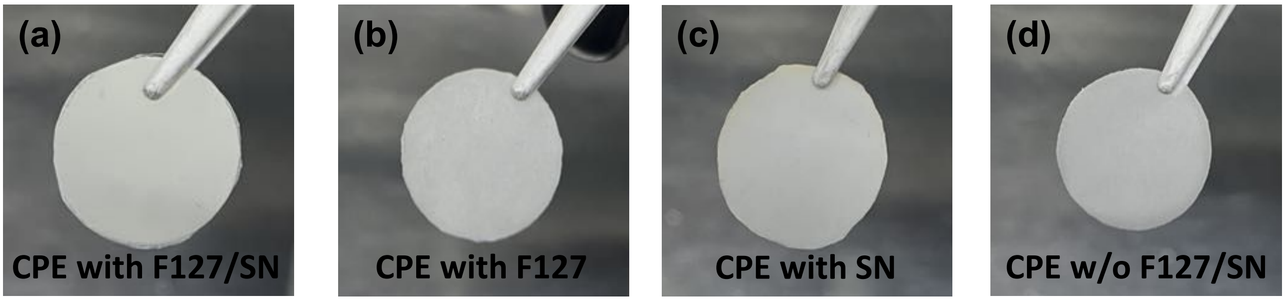

Figure S2 Optical images of different CPEs (a) CPE with F127/SN (b) CPE with F127 (c) CPE with SN (d) CPE w/o F127/SN

Figures S3a, b present the SEM morphology of the S@C composite synthesized via the hydrothermal method. The presence of S@C was confirmed by the XRD pattern shown in **Figure S3c**. Thermogravimetric analysis (TGA), as illustrated in **Figure S3d**, reveals a sulfur loading of approximately 68 wt% in the S@C composite. **Figures S3e, f** display the nitrogen adsorption–desorption isotherms and the corresponding pore size distribution of the S@C, respectively, indicating that the pores are predominantly mesoporous, with sizes mainly distributed in the range of 30 to 100 nm.

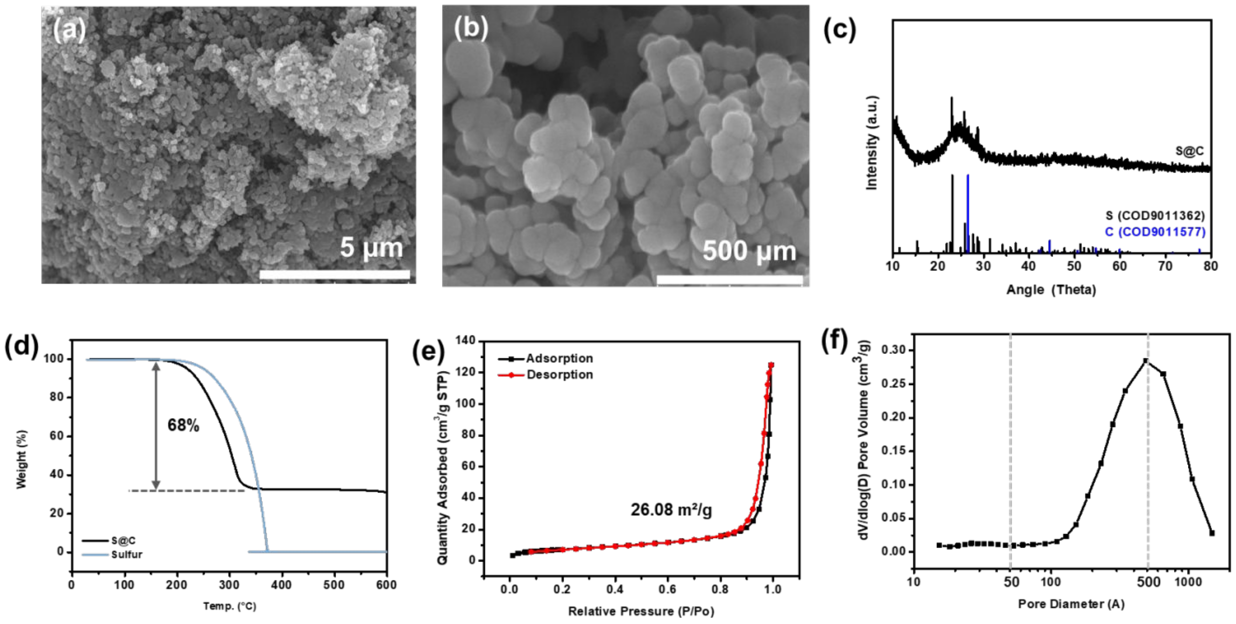

Figure S3 Cathode S@C material characterization (a), (b) SEM images, (C) XRD patten, (d) TGA thermogram, (e) N2 adsorption-desorption isotherm, (f) pore size distributions

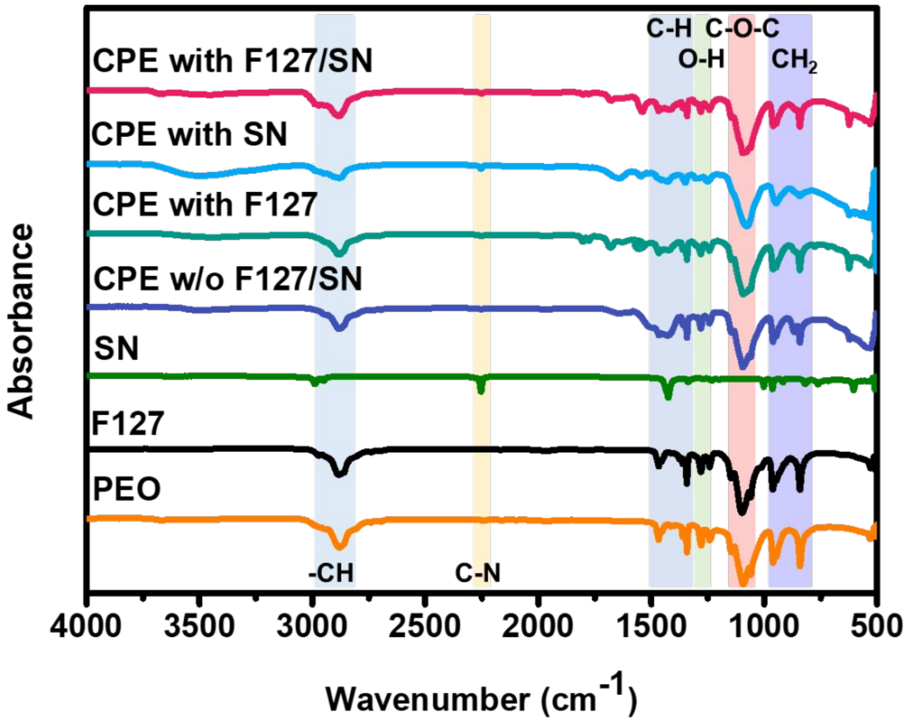

Figure S4 FTIR spectra of pure PEO, pure F127, Pure SN and different CPEs.

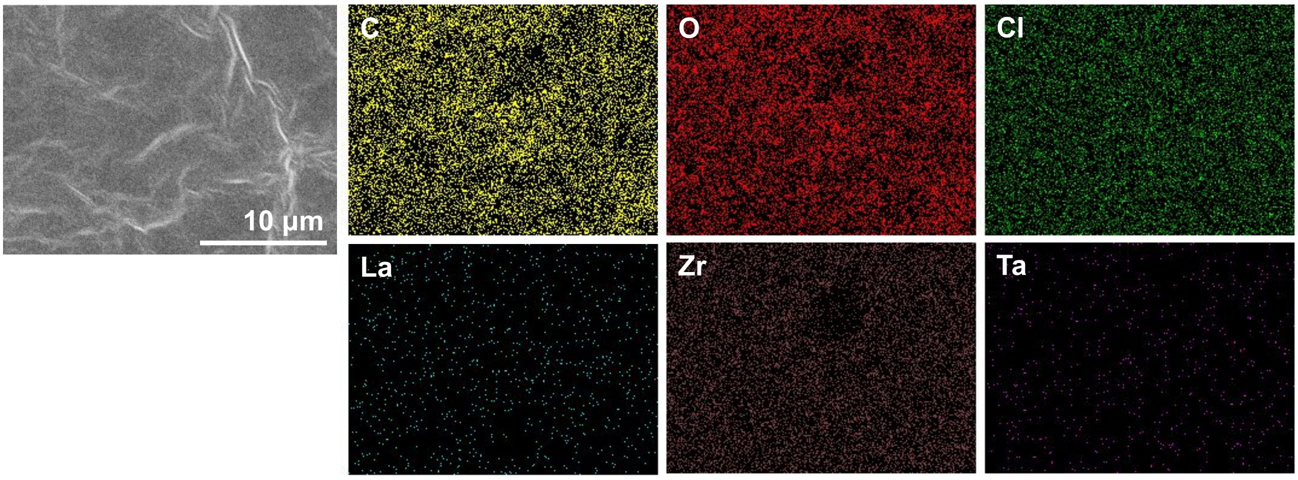

Figure S5 SEM image and EDS mapping of CPE with F127

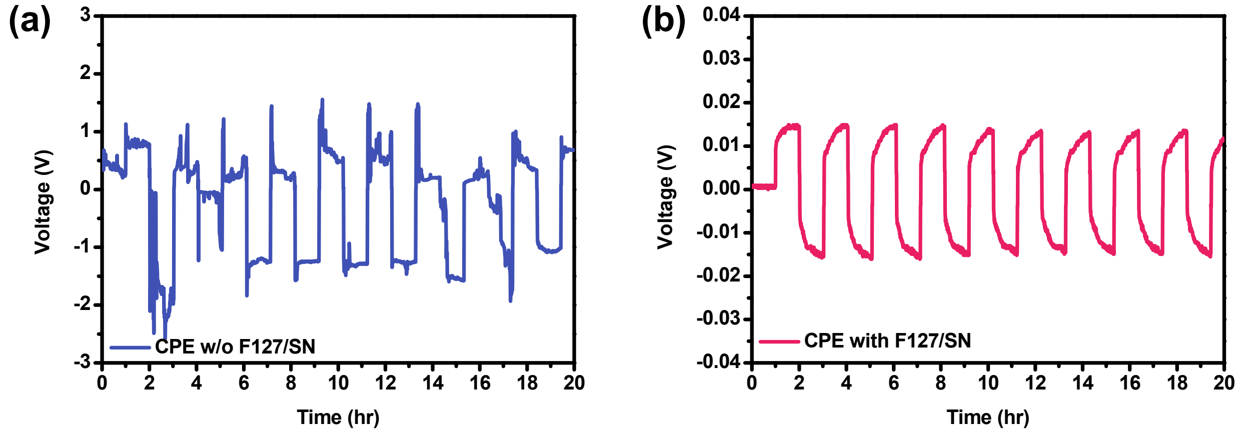

Figure S6 Enlarged galvanostatic cycling of (a) the CPE w/o F127/SN, (b) the CPE with F127/SN

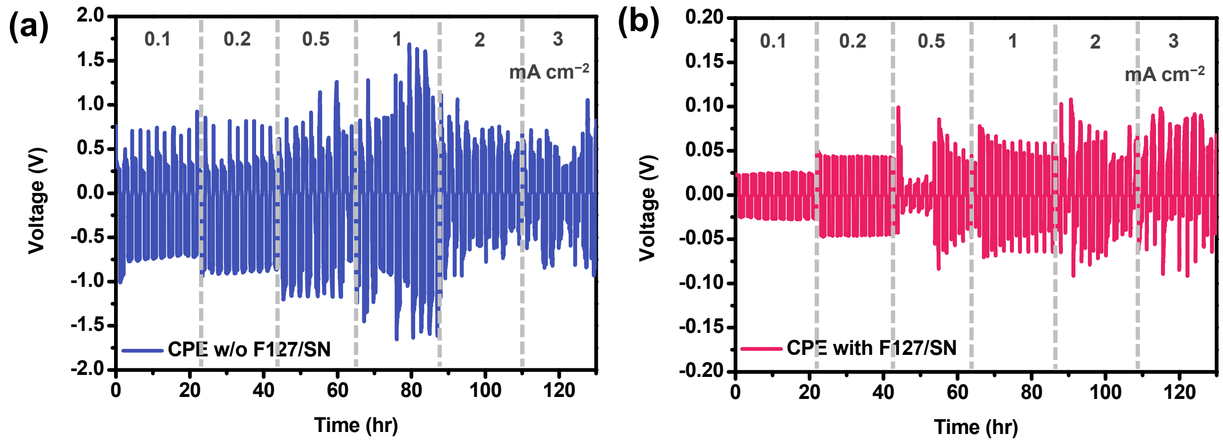

Figure S7 Galvanostatic cycling of the (a) CPE w/o F127/SN and (b) CPE with F127/SN at different current densities

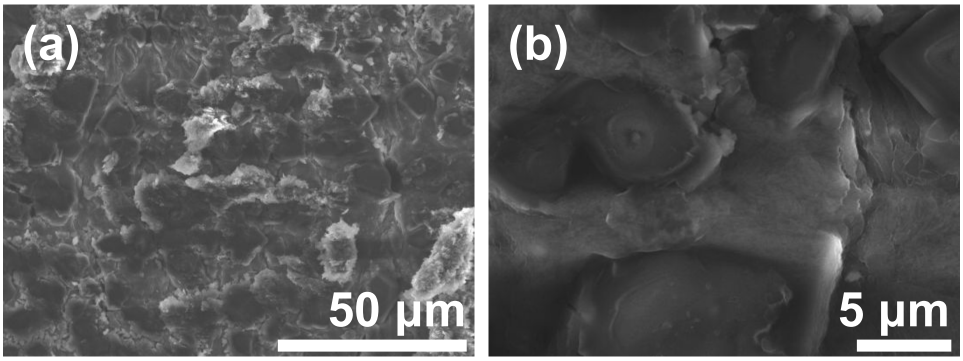

Figure S8 SEM images of the CPE with F127/SN after cycling at magnifications of (a) 1k (b) 5k

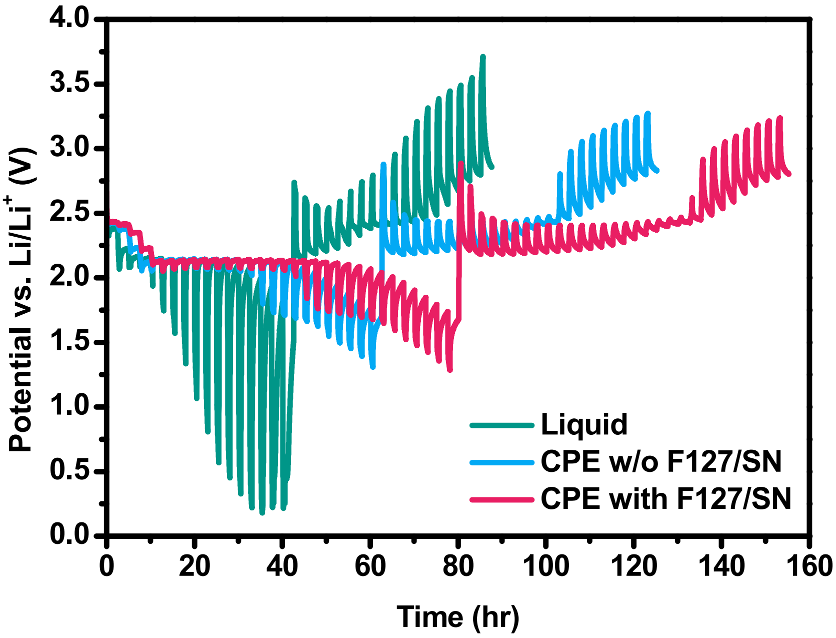

Figure S9 The comparison of GITT performance
